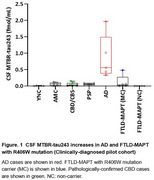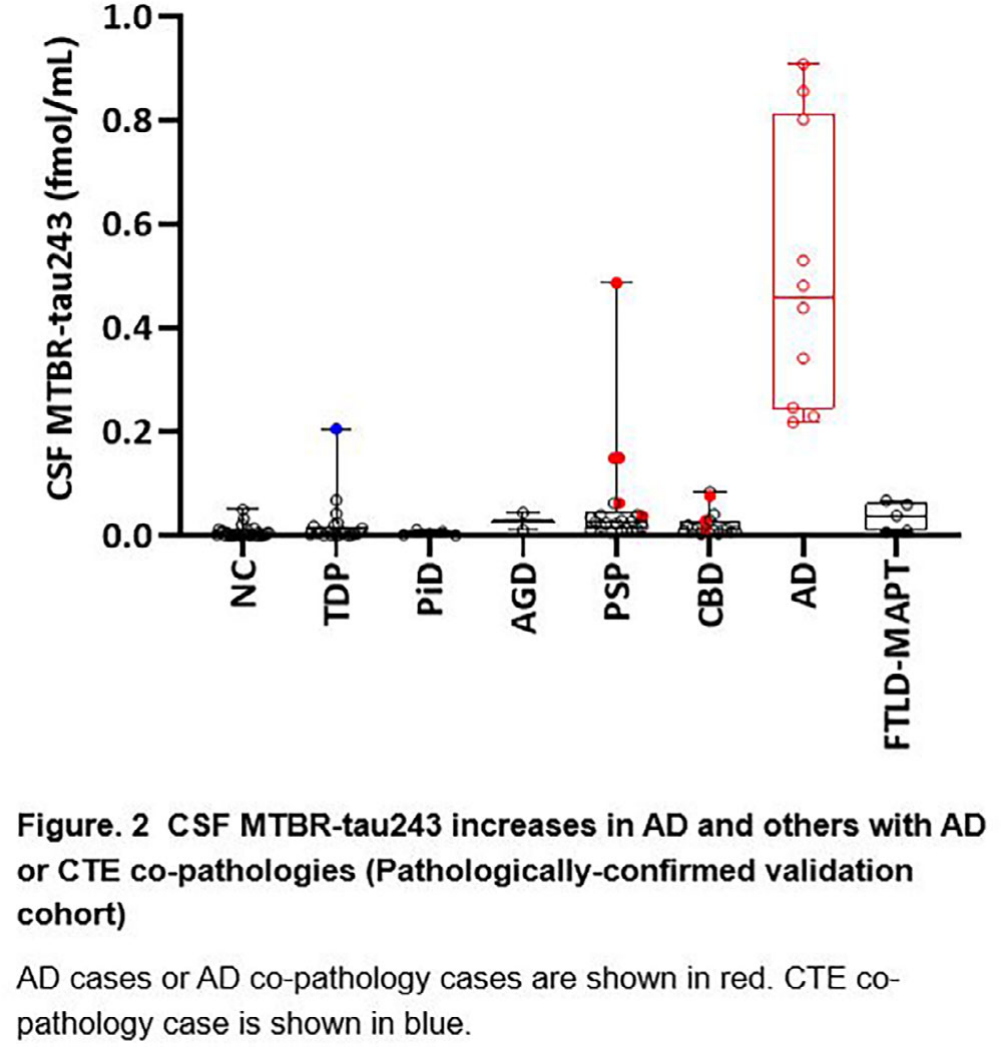# MTBR‐tau243 biomarker specifically identifies mixed 3R/4R tauopathies

**DOI:** 10.1002/alz70861_108254

**Published:** 2025-12-23

**Authors:** Kanta Horie, Rama Koppisetti, Salvatore Spina, Lawren VandeVrede, Ross W. Paterson, Nupur Ghoshal, Adam L. Boxer, Randall J. Bateman, Chihiro Sato

**Affiliations:** ^1^ Eisai Inc., Nutley, NJ USA; ^2^ Washington University School of Medicine, St. Louis, MO USA; ^3^ The Tracy Family SILQ Center, St. Louis, MO USA; ^4^ University of California San Francisco, San Francisco, CA USA; ^5^ UCL Queen Square Institute of Neurology, London UK

## Abstract

**Background:**

Insoluble tau aggregates are a defining neuropathological feature of Alzheimer's disease (AD), and primary tauopathies like corticobasal degeneration (CBD), progressive supranuclear palsy (PSP), Pick’s disease (PiD), and frontotemporal lobar degeneration secondary to MAPT mutations (FTLD‐MAPT). Recently, we developed assays that quantify tau microtubule‐binding region containing residue 243 (MTBR‐tau243) in AD cerebrospinal fluid (CSF) and blood plasma and found that MTBR‐tau243 in biofluids accurately predicts AD tau pathology as measured by tau‐PET than other tau species [1,2]. However, it is unknown whether the MTBR‐tau243 increases in AD specifically or in other tauopathies as well.

**Method:**

We quantified CSF MTBR‐tau243 by mass spectrometry in two cohorts: 1. Clinically‐diagnosed pilot cohort including 41 participants with corticobasal syndrome (CBS, n=9), PSP‐Richardson’s syndrome (PSP‐RS) (*n* =7), FTLD‐MAPT mutation carriers (*n* =5) and non‐carrier family members (*n* =5), AD (*n* =5, with positive amyloid‐PET) and cognitively‐normal controls (young‐normal control n=5, age‐matched control n=5) and 2. Pathologically‐confirmed validation cohort including 112 participants with CBD (*n* =18), PSP (*n* =22), FTLD‐MAPT (*n* =5), PiD (*n* =5), AGD (*n* =2), AD (*n* =10), FTLD with TDP‐43 proteinopathy (FTLD‐TDP) (*n* =21) and cognitively‐normal control (*n* =29).

**Result:**

CSF MTBR‐tau243 levels significantly increased in all AD participants and one FTLD‐MAPT with R406W mutation carrier while not in PSP and CBS (including two pathologically‐confirmed CBD cases) in the pilot clinically‐diagnosed cohort (Figure 1). The pathologically‐confirmed validation cohort reproduced the results that only AD or other participants with AD co‐pathology particularly increased MTBR‐tau243 in CSF. Interestingly, one outlier participant in FTLD‐TDP group exhibited the co‐pathology of chronic traumatic encephalopathy (CTE) which accumulates mixed 3R/4R tau aggregates in brain like AD and FTLD‐MAPT with R406W mutation (Figure 2).

**Conclusion:**

MTBR‐tau243 biomarker specifically reflects the accumulation of aggregated insoluble tau in mixed 3R/4R tauopathies including AD, CTE and FTLD‐MAPT with R406W mutation while not in 4R (CBD, PSP, AGD) or 3R primary tauopathies (PiD). MTBR‐tau243 biomarker can stage mixed 3R/4R tauopathies, aiding clinicians in determining the underlying cause of cognitive impairment and monitoring the efficacy of tau‐targeted therapies.

**Reference**:

[1] Horie, K., et al. *Nat Med* 29, 1954–1963 (2023).

[2] Horie, K., et al. *Nat Med* (2025). https://doi.org/10.1038/s41591‐025‐03617‐7